# Supplementary material for: Potential clinical relevance of cardiac magnetic resonance to diagnose cardiac light chain amyloidosis
Source: PLoS One. 2022 Jun 13;17(6):e0269807. doi: 10.1371/journal.pone.0269807 (PMC9191721; doi:10.1371/journal.pone.0269807)
Supplement: S1 Table — Intraclass correlation analysis for interobserver variability in strain parameters. (DOCX) [file pone.0269807.s001.docx]

**S1 Table. Reproducibility of stain analyses.** Intraclass correlation analysis for interobserver variability in strain parameters.

|  | Intraclass correlation | 95% Confidence interval |
| --- | --- | --- |
| GRS (%) | 0.94 | 0.89 to 0.96 |
| GCS (%) | 0.93 | 0.88 to 0.96 |
| GLS (%) | 0.93 | 0.88 to 0.96 |
| Delta rotation | 0.30 | -0.25 to 0.60 |
| SD-CS-Peak | 0.52 | 0.17 to 0.72 |
| SD-LS-Peak | 0.88 | 0.71 to 0.94 |
| MDC (%) | 0.72 | 0.50 to 0.84 |
| MDL (%) | 0.63 | 0.36 to 0.79 |
| basal CS (%) | 0.92 | 0.85 to 0.96 |
| mid CS (%) | 0.95 | 0.90 to 0.97 |
| apical CS (%) | 0.79 | 0.60 to 0.89 |
| basal LS (%) | 0.87 | 0.76 to 0.93 |
| mid LS (%) | 0.96 | 0.93 to 0.98 |
| apical LS (%) | 0.92 | 0.85 to 0.96 |
