# Supplementary material for: Potential clinical relevance of cardiac magnetic resonance to diagnose cardiac light chain amyloidosis
Source: PLoS One. 2022 Jun 13;17(6):e0269807. doi: 10.1371/journal.pone.0269807 (PMC9191721; doi:10.1371/journal.pone.0269807)
Supplement: S2 Table — Results of the ROC curve analyses. (DOCX) [file pone.0269807.s002.docx]

**S2 Table. Diagnostic accuracy of CMR parameters in differentiating CA from HCM and CA from controls with HT.**

Results of the ROC curve analyses.

|  | cardiac AL-amyloidosis vs. HCM | | | | |
| --- | --- | --- | --- | --- | --- |
|  | sensitivity | specificity | cut off | AUC | p |
| LVEF | 60% | 95% | 53% | 0.829 | <0.001 |
| LVESVi | 60% | 70% | 37 ml/m^2^ | 0.708 | <0.001 |
| LVSVi | 66% | 87% | 43 ml/m^2^ | 0.774 | <0.001 |
| max. EDWT | 94% | 41% | 20 mm | 0.634 | <0.001 |
| LGE% | 76% | 87% | 16% | 0.916 | <0.001 |
| GRS | 83% | 70% | 74% | 0.847 | <0.001 |
| GCS | 66% | 75% | -36% | 0.746 | <0.001 |
| GLS | 86% | 63% | -23% | 0.803 | <0.001 |
| SD-LS-Peak | 66% | 65% | 11 | 0.671 | <0.001 |
| basal CS | 71% | 83% | -31% | 0.874 | <0.001 |
| mid CS | 63% | 82% | -31% | 0.734 | <0.001 |
| apical CS | 71% | 54% | -47% | 0.653 | <0.01 |
| apex-to-base CS | 63% | 74% | 1.44 | 0.741 | <0.001 |
| basal LS | 69% | 85% | -16% | 0.847 | <0.001 |
| mid LS | 83% | 43% | -25% | 0.703 | <0.001 |
| apical LS | 60% | 79% | -24% | 0.731 | <0.001 |
| apex-to-base LS | 71% | 49% | 1.45 | 0.609 | <0.05 |
| GRS/EF | 83% | 64% | 1.26 | 0.8 | <0.001 |

|  | cardiac AL-amyloidosis vs. HT | | | | |
| --- | --- | --- | --- | --- | --- |
|  | sensitivity | specificity | cut off | AUC | p |
| LVSVi | 46% | 84% | 38 ml/m^2^ | 0.648 | <0.05 |
| LVMi | 100% | 74% | 61 g/m^2^ | 0.927 | <0.001 |
| max. EDWT | 94% | 89% | 14 mm | 0.967 | <0.001 |
| LGE% | 97% | 98% | 6% | 0.995 | <0.001 |
| GCS | 66% | 75% | -36% | 0.746 | <0.001 |
| GLS | 66% | 64% | -20% | 0.691 | <0.001 |
| MDC | 46% | 80% | 5,4% | 0.641 | <0.05 |
| MDL | 69% | 87% | 16.3% | 0.786 | <0.001 |
| mid CS | 66% | 67% | -28% | 0.654 | <0.05 |
| apical CS | 77% | 74% | -35% | 0.761 | <0.001 |
| apex-to-base CS | 77% | 80% | 1.24 | 0.781 | <0.001 |
| basal LS | 94% | 79% | -21% | 0.939 | <0.001 |
| mid LS | 51% | 91% | -20% | 0.771 | <0.001 |
| apical LS | 60% | 79% | -24% | 0.731 | <0.001 |
| apex-to-base LS | 83% | 76% | 1.17 | 0.860 | <0.001 |
| GLS/EF | 57% | 80% | -0,37 | 0.672 | <0.01 |
| GCS/EF | 69% | 94% | -0.59 | 0.824 | <0.001 |
