# Supplementary material for: Potential clinical relevance of cardiac magnetic resonance to diagnose cardiac light chain amyloidosis
Source: PLoS One. 2022 Jun 13;17(6):e0269807. doi: 10.1371/journal.pone.0269807 (PMC9191721; doi:10.1371/journal.pone.0269807)
Supplement: Attachment — Submitted filename: Response to Reviewers.docx [file pone.0269807.s004.docx]

**Cover letter, questions and our responses (the questions are shown in bold):**

Dear Daniel A. Morris,

Thank you for your positive response to our manuscript. Attached, please find our revised manuscript. We have answered the questions that the Editor and the Reviewer raised. We appreciate the helpful comments from the Editor and the Reviewer. We believe that the manuscript has been improved by incorporating these comments and recommendations.

Thank you very much for your consideration. We look forward to hearing your decision.

Best regards,

Hajnalka Vago

**Additional Editor Comments:

Thank you very much for submitting this large study to PlosOne. While the cohort and analyses performed are analyzed interesting, several pending serious and major limitations should be addressed in order to get adequate clinical applicability of the findings from this study.

Pending Major Limitations and Comments:**

**1) Concerns stated by the reviewer:
- The reviewer has addressed important limitations from this study, which should be mandatorily addressed in the revised version.**

Thank you for your comment. The limitations section has been completed with the followings:

“The main advantage of feature-tracking strain analysis is that it needs no additional dedicated CMR sequences, and the evaluation is performed using the standard cine images. However, this method has some limitations: previously published data showed that reliability and accuracy of feature-tracking analysis is dependent on reader experience more than tagging-based strain analysis, and the reproducibility of segmental assessment of strain is lower. There might be some referral bias that can explain the relatively low prevalence of CA patients, especially ATTR patients: ATTR patients are usually referred from community cardiac services or from other cardiology centers where diagnostic awareness is lower compared to hematology centers where myeloma and MGUS patients are monitored for CA and from where a great number of AL patients are referred. In recent years, cardiology centers have diagnosed TTR CA in a "non-biopsy" manner in many cases with the help of PYP scans. Many of these TTR CA pts did not have a CMR for the diagnosis.”

**3) Uncertainty of the clinical applicability of the LV strain parameters:
- As it is shown in table 2, the rate of false positives and false negatives varied from 20 to 40% between the diverse strain parameters, which obligates to research alternative parameters to accurately differentiate HCM from CA. Hence, the authors should further analyze the diagnostic performance of ECV, T1 native mapping time, and LV phenotype to mainly differentiate HCM from CA.**

Thank you for your comment. We agree with the Editor that ECV and T1 mapping parameters increase the diagnostic accuracy of CMR in the differentiation of CA from HCM. Unfortunately, mapping measurements were not available in our center at the time of the investigated examinations. It has been signed in the limitation section.

The discussion section has been completed with the followings: “In recent years, novel CMR techniques, such as mapping measurements, have been developed for the quantitative assessment of myocardial changes. CA is characterized by pronouncedly increased native T1 values. In the case of contrast administration, the extracellular volume of the myocardium can be evaluated with T1 mapping. An increase in extracellular volume is an early marker of CA even before the appearance of LGE. Unfortunately, mapping measurements were not available in our center for the current study.”

Regarding LV phenotype, we have completed the results section with the followings:

“The most common form of HCM was asymmetric hypertrophy with a septal or an anterior distribution, which was found in 257 patients (77.9%). There were 47 (14.2%) patients with apical HCM, 21 (6.4%) patients with concentric HCM and five (1.5%) patients with midventricular HCM. Among CA patients, concentric hypertrophy was found in 16 cases (35%), and hypertrophy showed septal dominance in 30 patients (65%).”

“There was no difference in LVMi between HCM and CA patients; however, HCM patients had higher EDWT. Concentric hypertrophy was more frequent among CA patients.”

**4) Lack of incremental value analyses:
- The authors should show the sensibility, specificity, accuracy of the following parameters or findings to differentiate HCM from CA:

- Mid Septal ECV > 0,40
- Mid Septal ECV > 0,30
- Mid septal T1 native mapping time > 1200 ms
- Mid septal T1 native mapping time > 1000 ms
- Septal and posterior wall thickness ≥ 15mm
- Septal wall thickness ≥ 25mm
- Septal wall thickness ≥ 15mm
- Septal wall thickness ≥ 14mm
- Septal wall thickness ≥ 12mm
- Septal and posterior diffuse subendocardial LGE
- Septal diffuse subendocardial LGE
- Septal and posterior myocardial nulling prior to blood pool nulling or difficulty in achieving myocardial nulling.
- Septal myocardial nulling prior to blood pool nulling or difficulty in achieving myocardial nulling.
- Apical sparing using LV global longitudinal strain (GLS) (i.e., ratio average apical segments to average basal segments > 2).
- GLS < 15% and apical sparing
- Septal and posterior wall thickness ≥ 14mm + GLS < 15% and apical sparing + pericardial effusion
- Septal and posterior wall thickness ≥ 14mm + GLS < 15% and apical sparing + RV free wall thickness ≥ 7mm
- Septal and posterior wall thickness ≥ 12mm + GLS < 15% and apical sparing + RV free wall thickness ≥ 5mm**

Thank you for your advice. We added the required parameters to a new table (except mapping values) and completed the results section as follows. However, the results of the performed ROC curve analyses showed other cut-off values for GLS (>-23) and apex-to-base LS ratio (>1.36), as recommended by the Editor. Therefor we presented data with our cut-off values as well.

“The frequencies of positive results of different diagnostic criteria in the patient groups are shown in Table 3. The presence of septal or septal and posterior diffuse subendocardial LGE had high specificity (99% and 99.4%, respectively) and relatively high sensitivity (89% for both). The specificity of myocardial nulling prior to blood pool nulling or difficulty in achieving myocardial nulling was 100%, with a sensitivity of 68%.”

**Table 3**

|  | HCM | CA | p |
| --- | --- | --- | --- |
| Septal and posterior wall thickness ≥ 15 mm (%) | 7 | 35 | <0.0001 |
| Septal wall thickness ≥ 25 mm (%) | 18 | 2 | <0.01 |
| Septal wall thickness ≥ 15 mm (%) | 81 | 94 | <0,05 |
| Septal wall thickness ≥ 14 mm (%) | 87 | 94 | 0.07 |
| Septal wall thickness ≥ 12 mm (%) | 96 | 98 | 0.46 |
| Septal and posterior diffuse subendocardial LGE (%) | 0.6 | 89 | <0.0001 |
| Septal diffuse subendocardial LGE (%) | 1 | 89 | <0.0001 |
| Septal and posterior myocardial nulling prior to blood pool nulling or difficulty in achieving myocardial nulling (%) | 0 | 68 | <0.0001 |
| Septal myocardial nulling prior to blood pool nulling or difficulty in achieving myocardial nulling (%) | 0 | 68 | <0.0001 |
| apical sparing (apex-to-base LS > 2) (%) | 20 | 37 | <0.01 |
| GLS > -15% and apical sparing (%) | 0 | 9 | <0.0001 |
| Septal and posterior wall thickness ≥ 14 mm + GLS > -15% and apical sparing + pericardial effusion (%) | 0 | 2 | <0.01 |
| Septal and posterior wall thickness ≥ 14 mm + GLS > -15% and apical sparing + RV free wall thickness ≥ 7 mm (%) | 0 | 2 | <0.01 |
| Septal and posterior wall thickness ≥ 12 mm + GLS > -15% and apical sparing + RV free wall thickness ≥ 5 mm (%) | 0 | 4 | <0.0001 |
| apex-to-base LS > 1,36 (%) | 57 | 80 | <0.01 |
| GLS > -23% and apex-to-base LS > 1,36 (%) | 19 | 65 | <0.0001 |
| Septal and posterior wall thickness ≥ 14 mm + GLS > -23% and apex-to-base LS > 1,36 + pericardial effusion (%) | 0.6 | 22 | <0.0001 |
| Septal and posterior wall thickness ≥ 14 mm + GLS > -23% and apex-to-base LS > 1,36 + RV free wall thickness ≥ 7 mm (%) | 0.6 | 6.5 | <0.001 |
| Septal and posterior wall thickness ≥ 12 mm + GLS > -23% and apex-to-base LS > 1,36 + RV free wall thickness ≥ 5 mm (%) | 3 | 17 | <0.0001 |

Frequencies of positive diagnostic criteria in the HCM and CA patient groups compared with the chi-squared test

**Journal requirements:**

**When submitting your revision, we need you to address these additional requirements.**

**1. Please ensure that your manuscript meets PLOS ONE's style requirements, including those for file naming. The PLOS ONE style templates can be found at**

<https://journals.plos.org/plosone/s/file?id=wjVg/PLOSOne_formatting_sample_main_body.pdf> and

<https://journals.plos.org/plosone/s/file?id=ba62/PLOSOne_formatting_sample_title_authors_affiliations.pdf>

We have updated the manuscript’s style according to PLOS ONE’s style requirements.

**2. In your Data Availability statement, you have not specified where the minimal data set underlying the results described in your manuscript can be found. PLOS defines a study's minimal data set as the underlying data used to reach the conclusions drawn in the manuscript and any additional data required to replicate the reported study findings in their entirety. All PLOS journals require that the minimal data set be made fully available. For more information about our data policy, please see** [**http://journals.plos.org/plosone/s/data-availability**](http://journals.plos.org/plosone/s/data-availability)**.**

**Upon re-submitting your revised manuscript, please upload your study’s minimal underlying data set as either Supporting Information files or to a stable, public repository and include the relevant URLs, DOIs, or accession numbers within your revised cover letter. For a list of acceptable repositories, please see** [**http://journals.plos.org/plosone/s/data-availability#loc-recommended-repositories**](http://journals.plos.org/plosone/s/data-availability#loc-recommended-repositories)**. Any potentially identifying patient information must be fully anonymized.**

**Important: If there are ethical or legal restrictions to sharing your data publicly, please explain these restrictions in detail. Please see our guidelines for more information on what we consider unacceptable restrictions to publicly sharing data:** [**http://journals.plos.org/plosone/s/data-availability#loc-unacceptable-data-access-restrictions**](http://journals.plos.org/plosone/s/data-availability#loc-unacceptable-data-access-restrictions)**. Note that it is not acceptable for the authors to be the sole named individuals responsible for ensuring data access.**

**We will update your Data Availability statement to reflect the information you provide in your cover letter**

We have uploaded our study’s minimal underlying data set as Supporting Information file.

**3. Thank you for stating the following in the Funding Section of your manuscript:**

**“Project no. NVKP_16-1–2016-0017 (’National Heart Program’) has been implemented with the support provided from the National Research, Development and Innovation Fund of Hungary, financed under the NVKP_16 funding scheme. The research was financed by the Thematic Excellence Programme (2020-4.1.1.-TKP2020) of the Ministry for Innovation and Technology in Hungary, within the framework of the Therapeutic Development and Bioimaging thematic programmes of the Semmelweis University; and by the Ministry of Innovation and Technology NRDI Office within the framework of the Artificial Intelligence National Laboratory Program. LS was supported by the ÚNKP-20-3-II-SE-61 New National Excellence Program of the Ministry for Innovation and Technology from the source of the National Research, Development and Innovation Fund. ZD and LS were supported by the „Development of scientific workshops of medical, health sciences and pharmaceutical educations” project. Project identification number: EFOP-3.6.3-VEKOP-16-2017-00009.**

**We note that you have provided additional information within the Funding Section. Please note that funding information should not appear in other areas of your manuscript. We will only publish funding information present in the Funding Statement section of the online submission form.**

**Please remove any funding-related text from the manuscript and let us know how you would like to update your Funding Statement. Currently, your Funding Statement reads as follows:**

**“Project no. NVKP_16-1–2016-0017 (’National Heart Program’) has been implemented with the support provided from the National Research, Development and Innovation Fund of Hungary, financed under the NVKP_16 funding scheme. The research was financed by the Thematic Excellence Programme (2020-4.1.1.-TKP2020) of the Ministry for Innovation and Technology in Hungary, within the framework of the Therapeutic Development and Bioimaging thematic programmes of the Semmelweis University; and by the Ministry of Innovation and Technology NRDI Office within the framework of the Artificial Intelligence National Laboratory Program. LS was supported by the ÚNKP-20-3-II-SE-61 New National Excellence Program of the Ministry for Innovation and Technology from the source of the National Research, Development and Innovation Fund. ZD and LS were supported by the „Development of scientific workshops of medical, health sciences and pharmaceutical educations” project. Project identification number: EFOP-3.6.3-VEKOP-16-2017-00009. The funders had no role in study design, data collection and analysis, decision to publish, or preparation of the manuscript.”**

**Please include your amended statements within your cover letter; we will change the online submission form on your behalf.**

We have removed funding-related text from the manuscript. We have not changed our Funding Statement.

**4. Please include captions for your Supporting Information files at the end of your manuscript, and update any in-text citations to match accordingly. Please see our Supporting Information guidelines for more information:** [**http://journals.plos.org/plosone/s/supporting-information**](http://journals.plos.org/plosone/s/supporting-information)**.**

We have included captions for the Supporting Information file at the end of the manuscript.

**5) In order to refine and focus the findings, please exclude patients with Fabry disease and endomyocardial fibrosis.**

Thank you for your advice. We excluded patients with Fabry disease and endomyocardial fibrosis from the study.

**Reviewers' comments:**

Please use the space provided to explain your answers to the questions above. You may also include additional comments for the author, including concerns about dual publication, research ethics, or publication ethics. (Please upload your review as an attachment if it exceeds 20,000 characters)

**Author comment:**

We would like to thank the reviewer for the thorough reading of our manuscript and the constructive suggestions. We believe that the manuscript has been improved by incorporating these comments and recommendations.

**Reviewer #1:**

In the present paper Dohy and Merkely et al investigate the ability of myocardial strain by feature tracking analysis for the diagnostic classification and risk stratification of patients with myocardial hypertrophy due to HCM, cardiac amyloidosis (AL and ATTR), Fabry disease and endomyocardial fibrosis. Myocardial strain variables were able to differentiate between HCM and cardiac amyloidosis, whereas risk stratification could also be provided. The paper is surely of interest since strain is an important issue with cardiac imaging and the differentiation between diseases causing hypertrophy has nowadays important therapeutic implications for the specific management of such patients more than ever. However, some specific points definitely need to be clarified.

**1. The percentage of patients with HCM is strikingly high compared to those with cardiac amyloidosis. Especially ATTR patients are expected to be found much more frequently in a tertiary setting and AL patients in conjunction with hematology / oncology units. Please explain and consider reporting / acknowledging referral biases.**

**2. In the same direction, the number of ATTR patients is strikingly low with 3 patients in total! This is certainly not in agreement with current trends of diagnosis this entity in 30-fold more patients than in the last decade. The prevalence especially in elderly patients with hypertrophy of unknown origin is very high, if correctly diagnosed based on current algorithms.**

Thank you for your questions.

Answer for question 1 and 2:

There was certainly some referral bias. Myeloma and MGUS pts are monitored for CA in hematology centers, from where a great number of AL patients are referred. In other words, hematologists have a high awareness of AL. For them, CMR is ideal for the diagnosis of CA.

On the other hand, ATTR patients are usually referred from community cardiac services or from other cardiology centers where diagnostic awareness is lower (see below). In recent years, these centers have diagnosed TTR CA in a "non-biopsy" manner in many cases with the help of PYP scans. Many of these TTR CA pts did not have a CMR for the diagnosis. Another possible explanation for the relatively low number of ATTRwt patients is that the expected life expectancy in Hungary is significantly lower than that in Western Europe and the US, and therefore fewer patients "live long enough" to obtain ATTRwt. Additionally, the severe comorbidity of the elderly population in Hungary is quite high; therefore, we speculate that CMR is not performed in many cases, as it would not change the disease course.

In a very large series of systemic amyloid (not only CA) pts, Wechalekar et al published in 2016 in the Lancet that the ratio of AL was stable at approximately 67% over the past decades. The ratio of ATTR was growing simultaneously with the lowering number of AAs. In our retrospective study, the cause of CA remained unknown in 7 pts, but probably some of them also had ATTR. All together our data, i.e., the ratio of AL and ATTR, are not far from the data of Wechalekar et al.

CA is considered to be a rare disease, and diagnostic awareness is still low in Hungary. It is nicely shown by a recent paper which found that the prevalence of ATTRv is just half of the prevalence observed in non-endemic regions of Western Europe, although the number of recognized cases is increasing. (Pozsonyi et al, 2021, Genes). This is also one cause, which explains the higher ratio of HCM and lower CA patients among LVH morphology pts in Hungary.

We have completed the limitations sections with the followings: “There might be some referral bias that can explain the relatively low prevalence of CA patients, especially ATTR patients: ATTR patients are usually referred from community cardiac services or from other cardiology centers where diagnostic awareness is lower compared to hematology centers where myeloma and MGUS patients are monitored for CA and from where a great number of AL patients are referred. In recent years, cardiology centers have diagnosed TTR CA in a "non-biopsy" manner in many cases with the help of PYP scans. Many of these TTR CA pts did not have a CMR for the diagnosis.”

**3. In the same line the authors did not include patients with hypertensive heart disease, where in many cases differential diagnosis regarding HCM and cardiac amyloidosis may be very challenging, especially in those with progressed hypertensive heart disease, where focal LGE may be present, mimicking HCM. This needs to be explained by the authors, since inclusion of such patients would have been clinically meaningful and since inclusion of patients has performed retrospectively, which means that such patients could have been easily included and considered for feature tracking analysis too.**

Thank you for your comment. Differentiating hypertensive heart disease from HCM is highly clinically relevant. Patients with left ventricular hypertrophy obviously due to untreated hypertension are usually not referred to CMR in our center. Patients with hypertensive heart disease are usually referred for CMR if there is a suspicion of other underlying conditions behind LV hypertrophy. In these cases, differentiating hypertensive heart disease from mild phenotypic HCM is challenging. To avoid diagnostic uncertainty, we decided to exclude these patients from the study. As we involved in the study referred patients with clinical questions, we had only a few cases of LV hypertrophy certainly due to hypertensive heart disease.

**4. The same restrictions also apply for individuals with athletes’ heart, while in this case referral may be an issue.**

Thank you for your comment. We agree with the reviewer that differentiating athletes’ heart and pathological hypertrophy is crucial. As our clinic is a sports cardiology center, we perform CMR examinations in athletes in a large number. Nine athletes who were diagnosed with HCM were involved in the study. Athletes with physiological sport adaptation were not investigated in the current study.

We completed the methods section with the followings: “Patients with untreated hypertension, significant aortic stenosis, or athletes with left ventricular hypertrophy due to physiological sport adaptation were not involved in the study.”

**5. Figure 1. It is striking that patients with hypertensive heart disease are not included. Please see also my comment #3.**

Thank you for your comment. According to our inclusion criteria, patients with hypertensive heart disease were not included in the study.

**6. Figure 2. It would be meaningful to parallel show the images of a normal volunteer for comparison to this cardiac amyloidosis patient with very progressed myocardial disease.**

Thank you for your advice. We added to Figure 2 the LGE images and the bull’s eye with segmental strain values of a patient without structural heart disease.

**7. Diagnosis of HCM. What about patients with septal wall thickness >13mm and family history of HCM?**

Thank you for your question. The diagnosis of HCM in first-degree relatives of patients with HCM was based on the presence of otherwise unexplained increased wall thickness ≥13 mm. We have completed the methods section with this information. HCM in the family history was known in 24 cases, and only 4 of them had a wall thickness of 13-14 mm.

**8. I am not sure if inclusion of patients with endomyocardial fibrosis is meaningful since hypertrophy is not necessarily associated with this entity. Possibly some overlap with hypertensive heart disease needs to be considered with these patients more than with other categories.**

Thank you for your comment. On the advice of the Editor, patients with EMF or Fabry disease have been excluded from the study.

**9. The differential diagnosis of HCM versus cardiac amyloidosis is clinical meaningful and a strength of the article. It is correct to highlight this in the results, as shown in Table 2. Hereby, LGE seems to be the stronger predictor, which can be expected. However, the value of strain is also relevant since basal LS und CS also provide relatively high AUC values.**

Thank you for your comment. We have completed this section with the followings:

“The frequencies of positive results of different diagnostic criteria in the patient groups are shown in Table 3. The presence of septal or septal and posterior diffuse subendocardial LGE had high specificity (99% and 99.4%, respectively) and relatively high sensitivity (89% for both). The specificity of myocardial nulling prior to blood pool nulling or difficulty in achieving myocardial nulling was 100%, with a sensitivity of 68%.”

**Table 3**

|  | HCM | CA | p |
| --- | --- | --- | --- |
| Septal and posterior wall thickness ≥ 15 mm (%) | 7 | 35 | <0.0001 |
| Septal wall thickness ≥ 25 mm (%) | 18 | 2 | <0.01 |
| Septal wall thickness ≥ 15 mm (%) | 81 | 94 | <0,05 |
| Septal wall thickness ≥ 14 mm (%) | 87 | 94 | 0.07 |
| Septal wall thickness ≥ 12 mm (%) | 96 | 98 | 0.46 |
| Septal and posterior diffuse subendocardial LGE (%) | 0.6 | 89 | <0.0001 |
| Septal diffuse subendocardial LGE (%) | 1 | 89 | <0.0001 |
| Septal and posterior myocardial nulling prior to blood pool nulling or difficulty in achieving myocardial nulling (%) | 0 | 68 | <0.0001 |
| Septal myocardial nulling prior to blood pool nulling or difficulty in achieving myocardial nulling (%) | 0 | 68 | <0.0001 |
| apical sparing (apex-to-base LS > 2) (%) | 20 | 37 | <0.01 |
| GLS > -15% and apical sparing (%) | 0 | 9 | <0.0001 |
| Septal and posterior wall thickness ≥ 14 mm + GLS > -15% and apical sparing + pericardial effusion (%) | 0 | 2 | <0.01 |
| Septal and posterior wall thickness ≥ 14 mm + GLS > -15% and apical sparing + RV free wall thickness ≥ 7 mm (%) | 0 | 2 | <0.01 |
| Septal and posterior wall thickness ≥ 12 mm + GLS > -15% and apical sparing + RV free wall thickness ≥ 5 mm (%) | 0 | 4 | <0.0001 |
| apex-to-base LS > 1,36 (%) | 57 | 80 | <0.01 |
| GLS > -23% and apex-to-base LS > 1,36 (%) | 19 | 65 | <0.0001 |
| Septal and posterior wall thickness ≥ 14 mm + GLS > -23% and apex-to-base LS > 1,36 + pericardial effusion (%) | 0.6 | 22 | <0.0001 |
| Septal and posterior wall thickness ≥ 14 mm + GLS > -23% and apex-to-base LS > 1,36 + RV free wall thickness ≥ 7 mm (%) | 0.6 | 6.5 | <0.001 |
| Septal and posterior wall thickness ≥ 12 mm + GLS > -23% and apex-to-base LS > 1,36 + RV free wall thickness ≥ 5 mm (%) | 3 | 17 | <0.0001 |

Frequencies of positive diagnostic criteria in the HCM and CA patient groups compared with the chi-squared test

We have highlighted these results in the discussion section:

“In the differentiation of CA from HCM, the amount and pattern of LGE have the highest diagnostic accuracy, followed by basal LS, basal CS and GRS. (…) In our study population, it was found that the amount and pattern of LGE had the highest diagnostic accuracy in the differentiation of CA and HCM. However, in case of a contraindication for contrast agent administration, further diagnostic methods are needed. We found that strain parameters have relatively high diagnostic accuracy. The sensitivity of GLS with a cut-off of -23% was 89%, while the specificities of basal LS (cut-off of -16%), basal CS (cut-off of -31%) and GRS (cut-off of 63%) were 85%, 83% and 83%, respectively.”

**10. Figure 4 demonstrates that patients with amyloidosis have poorer prognosis, which is expected due to the entity of the disease. However, much more meaningful would be a Kaplan-Maier analysis based on LGE and on strain values. Would one of the myocardial strain values be able to show prognostic relevance. If yes, this would surely contribute to the current literature.**

Thank you for your advice. Using ROC curve analysis, we calculated cut-off values of LGE, LVSVi, GLS, GCS and GRS regarding mortality. The survival probability of patient groups defined by the calculated cut-off parameters was analyzed and illustrated with Kaplan-Meier curves, which can be found in the revised Figure 4.

**11. In the same direction, basal LS seems to bear independent prognostic implications for the estimation of mortality. This needs to be tested and adjusted for quantitative LGE and main diagnosis. The corresponding Kaplan-Maier analysis needs to be demonstrated.**

Thank you for your comment. Our study population changed, as we excluded patients with EMF or Fabry disease from the analysis on the advice of the Editor. Furthermore, we completed the regional strain analysis of all HCM patients (in the original manuscript, regional strain analysis was performed on 89 randomly selected HCM patients). These factors led to a changed result of the survival analysis: independent predictors of mortality with multivariable Cox proportional hazard regression are a diagnosis of CA, age and GLS. Variables with p<0.05 in the univariable analysis were analyzed in one multivariable model (after excluding highly correlated predictors). As quantitative LGE and the main diagnosis were included in this model, the prognostic significance of GLS is independent of these factors. We believe this result is more reliable than the previous one was with a smaller patient group.

Kaplan-Meier curves based on LGE, LVSVi, GLS, GCS and GRS have been added to Figure 4.

**12. A recent study investigating the ability of strain based CMR (with fast-SENC) to differentiate between patients with HCM, amyloidosis and hypertensive heart disease needs to be reported and discussed by the authors (Giusca et al, JCMR 2021).**

Thank you for your comment. We have added the required article to the references and completed the discussion section with the followings: “A recently published study investigated the ability of a single heartbeat fast-strain encoded (SENC) CMR-derived myocardial strain to discriminate between CA, HCM, hypertensive heart disease, athletes’ heart and healthy controls. CA patients had the most impaired GLS and GCS values, and the percentage of LV segments with a strain value < -17% was the lowest in this patient group; apical sparing was not investigated.”

**13. In addition, some limitations need to be reported with feature tracking acquisitions. Although, a big advantage of feature tracking is no need for additional dedicated CMR sequences, some disadvantages have been extensively mentioned in the recent literature, especially regarding the segmental assessment of strain (Feisst A et al, IJC Heart Vasc. 2018; Mangion K et al, Sci Rep. 2019 and Almitairi HM et al, Br. J. Radiol. 2017).**

Thank you for your comment. We have completed the limitations section as follows: “The main advantage of feature-tracking strain analysis is that it needs no additional dedicated CMR sequences, and the evaluation is performed using the standard cine images. However, this method has some limitations: previously published data showed that reliability and accuracy of feature-tracking analysis is dependent on reader experience more than tagging-based strain analysis, and the reproducibility of segmental assessment of strain is lower.”
