# Supplementary material for: Potential clinical relevance of cardiac magnetic resonance to diagnose cardiac light chain amyloidosis
Source: PLoS One. 2022 Jun 13;17(6):e0269807. doi: 10.1371/journal.pone.0269807 (PMC9191721; doi:10.1371/journal.pone.0269807)
Supplement: Attachment — Submitted filename: Response to Reviewers.docx [file pone.0269807.s005.docx]

**Cover letter, questions and our responses (the questions are shown in bold):**

Dear Daniel A. Morris,

Thank you for your response to our manuscript. Attached, please find our revised manuscript. We have answered the questions that the Editor raised. We appreciate the helpful comments from the Editor. We believe that the manuscript has been improved by incorporating these comments and recommendations.

Thank you very much for your consideration. We look forward to hearing your decision.

Best regards,

Hajnalka Vago

Editor Comments :

I have read and revised again this interesting large study and appreciated the effort of the authors to address the suggestions of the editors and reviewers. While the data is interesting, there are pending major and serious limitations in this study, mainly regarding the clinical relevance, applicability, and presentation of this study.
Hence, without addressing the below detailed major limitations, this study will have a low priority for publication in PlosOne.

Major and Serious Pending Limitations:

1) Presentation of the Manuscript and Results:

**- The most important and clinically relevant findings from this study are those linked to AL-Amyloidosis (i.e., the sensitivity and specificity of some parameters to detect a cardiac involvement in this systematic hematological disease). In fact, while patients with cardiac ATTR-Amyloidosis are easily diagnosed with bone scintigraphy (planar and/or SPECT), the cardiac involvement in AL-Amyloidosis is challenging (i.e., SPECT does not provide any diagnostic help). In addition, the role of CMR in AL-Amyloidosis remains uncertain. Hence, the authors should make focus in patients with AL-Amyloidosis.**

Thank you for your comment. We have focused on patients with AL-Amyloidosis and have excluded patients with TTR or AA amyloidosis or if the exact type of amyloidosis was unknown. **- In line with the above-mentioned comments, it will necessary to include a control group of at least 70 patients (i.e., at least match 1:2) with arterial hypertension without history of cardiomyopathy and with similar LVEF than the group with AL-Amyloidosis. Including merely at least 70 control patients it would not mean a lot of effort in a normal CMR department.**

Thank you for your advice. Hypertensive heart disease patients are usually not referred for CMR in our Center if there is no suspicion of cardiomyopathy. We can retrospectively collect patients, who have in their patient’s history arterial hypertension (mostly treated hypertension) and were referred for CMR with an other indication but CMR found no structural heart disease. However, these patients usually have no expressed hypertrophy as their hypertension is treated. If the Editor suggest that we could increase the scientific value of our study with analyzing this patient population, we will complete our study with pleasure. **- Patients with other type of Amyloidosis other than AL should be excluded in order to get a homogenous and clinically relevant population.**

Thank you for your advice. We have excluded patients with TTR or AA amyloidosis and if the exact type of amyloidosis was unknown.

**- The most important analyses and parameters to analyze are the sensibility and specificity of CMR parameters to determine cardiac involvement as compared to controls patients and also to differentiate from CMH. Hence, the authors should mandatorily analyze and show the sensibility and specificity of the following parameters to determine cardiac Al-Amyloidosis (namely, in 2 separated tables, one AL-Amyloidosis vs. arterial hypertension; and another Al-Amyloidosis vs. CMH):
Septal and posterior wall thickness ≥ 15mm
Septal and posterior wall thickness ≥ 14mm
Septal and posterior wall thickness ≥ 13mm
Septal and posterior wall thickness ≥ 12mm
Septal wall thickness ≥ 20mm
Septal wall thickness ≥ 18mm
Septal wall thickness ≥ 15mm
Septal wall thickness ≥ 14mm
Septal wall thickness ≥ 13mm
Septal wall thickness ≥ 12mm
Septal and posterior diffuse subendocardial LGE
Septal diffuse subendocardial LGE
Septal and posterior myocardial nulling prior to blood pool nulling or difficulty in achieving myocardial nulling
Septal myocardial nulling prior to blood pool nulling or difficulty in achieving myocardial nulling
Apical sparing using LV global longitudinal strain (GLS) (i.e., ratio average apical segments to average basal segments > 2).
GLS < 15% and apical sparing
GLS < 15%
GLS < 13%
GLS < 12%**

Thank you for your advice. We have added a table with the sensitivity and specificity of the required parameters to differentiate CA from HCM:

|  | CA vs. HCM | |
| --- | --- | --- |
|  | sensitivity | specificity |
| Septal and posterior wall thickness ≥ 15 mm (%) | 29 | 93 |
| Septal and posterior wall thickness ≥ 14 mm (%) | 31 | 88 |
| Septal and posterior wall thickness ≥ 13 mm (%) | 37 | 81 |
| Septal and posterior wall thickness ≥ 12 mm (%) | 57 | 31 |
| Septal wall thickness ≥ 20 mm (%) | 9 | 60 |
| Septal wall thickness ≥ 18 mm (%) | 34 | 46 |
| Septal wall thickness ≥ 15 mm (%) | 91 | 19 |
| Septal wall thickness ≥ 14 mm (%) | 91 | 13 |
| Septal wall thickness ≥ 13 mm (%) | 94 | 9 |
| Septal wall thickness ≥ 12 mm (%) | 97 | 5 |
| Septal and posterior diffuse subendocardial LGE (%) | 88 | 99 |
| Septal diffuse subendocardial LGE (%) | 88 | 99 |
| Septal and posterior myocardial nulling prior to blood pool nulling or difficulty in achieving myocardial nulling (%) | 71 | 100 |
| Septal myocardial nulling prior to blood pool nulling or difficulty in achieving myocardial nulling (%) | 71 | 100 |
| apical sparing (apex-to-base LS > 2) (%) | 31 | 80 |
| GLS > -15% and apical sparing (%) | 6 | 100 |
| GLS > -15% | 23 | 93 |
| GLS > -13% | 17 | 96 |
| GLS > -12% | 9 | 96 |

**- Please provide the following cases examples:
1- AL-Amyloidosis with septal and posterior myocardial nulling prior to blood pool nulling or difficulty in achieving myocardial nulling
2- Arterial hypertension without septal and posterior myocardial nulling prior to blood pool nulling or difficulty in achieving myocardial nulling
3- CMH without septal and posterior myocardial nulling prior to blood pool nulling or difficulty in achieving myocardial nulling
4- AL-Amyloidosis with septal and posterior diffuse subendocardial LGE
5- Arterial hypertension without septal and posterior diffuse subendocardial LGE
6- CMH without septal and posterior diffuse subendocardial LGE**

Thank you for your comment. We provided examples of CA with myocardial nulling prior to blood pool nulling and diffuse subendocardial LGE, and a case of HCM with patchy mid-myocardial LGE in Figure 3. **- The outcome data is not of high relevance in this study, but if you consider important this data, please analyze only the mortality for HF or hospitalization for HF and in separated groups (i.e., those with CMH, AL-Amyloidosis, and arterial hypertension).**

Thank you for your advice. We have no information about the hospitalization in most of the cases, and also the cause of death is unknown in a part of the cases. Therefore, we decided to leave out the Cox regression analysis for the assessment the prognostic value of CMR parameters.

The Kaplan-Meier analyses are included the study later on, as it was the request of the Reviewer.
