# Supplementary material for: Potential clinical relevance of cardiac magnetic resonance to diagnose cardiac light chain amyloidosis
Source: PLoS One. 2022 Jun 13;17(6):e0269807. doi: 10.1371/journal.pone.0269807 (PMC9191721; doi:10.1371/journal.pone.0269807)
Supplement: Attachment — Submitted filename: Response to Reviewers.docx [file pone.0269807.s006.docx]

Additional Editor Comments:

I would like to congratulate to the authors for the effort made to improve the manuscript following the suggestions of the Editors and Reviewers. In fact, the presentation of the study has significantly improved and it remains just some limitations to be addressed to get the final version of this interesting study.

Minor Pending Limitations:

**1) It will absolutely necessary to include a control group of at least 70 patients (i.e., at least match 1:2) with arterial hypertension without history of cardiomyopathy and with similar LVEF than the group with AL-Amyloidosis.**

Thank you for your suggestion. We have added 70 patients with arterial hypertension to the study and analyzed the differential diagnostic significance of CMR parameters in CA patients vs. those with hypertension. We believe that the clinical relevance of our study increased with these findings. **2) Please a table as table 3, comparing the sensibility and specificity of the parameters of table but comparing AL-Amyloidosis vs. control patients with arterial hypertension.**

Thank you for your suggestion. We have added a table with the sensitivity and specificity of CMR parameters to differentiate CA from controls with HT:

|  | CA vs. HT | | |
| --- | --- | --- | --- |
|  | sensitivity | specificity | AUC |
| Septal and posterior EDWT ≥ 15 mm | 29% | 100% | 0.645 |
| Septal and posterior EDWT ≥ 14 mm | 31% | 100% | 0.655 |
| Septal and posterior EDWT ≥ 13 mm | 37% | 100% | 0.685 |
| Septal and posterior EDWT ≥ 12 mm | 57% | 97% | 0.770 |
| Septal EDWT ≥ 20 mm | 9% | 100% | 0.545 |
| Septal EDWT ≥ 18 mm | 34% | 100% | 0.670 |
| Septal EDWT ≥ 15 mm | 91% | 89% | 0.900 |
| Septal EDWT ≥ 14 mm | 91% | 83% | 0.870 |
| Septal EDWT ≥ 13 mm | 94% | 73% | 0.835 |
| Septal EDWT ≥ 12 mm | 97% | 54% | 0.755 |
| Septal and posterior diffuse subendocardial LGE | 88% | 100% | 0.940 |
| Septal diffuse subendocardial LGE | 88% | 100% | 0.940 |
| Septal and posterior myocardial nulling prior to blood pool nulling or difficulty in achieving myocardial nulling | 71% | 100% | 0.855 |
| Septal myocardial nulling prior to blood pool nulling or difficulty in achieving myocardial nulling | 71% | 100% | 0.855 |
| apical sparing (apex-to-base LS > 2) | 31% | 99% | 0.650 |
| GLS > -15% and apical sparing | 6% | 100% | 0.530 |
| GLS > -15% | 23% | 93% | 0.580 |
| GLS > -13% | 17% | 97% | 0.570 |
| GLS > -12% | 9% | 99% | 0.540 |

**3) Table 2 is not interesting and thus, it should be moved to data supplement.**

Thank you for your advice. We have moved the results of ROC analysis to data supplement. **4) The section on prognosis is interesting, but out of the scope of the present study. Hence, it should be removed, but it would be very interesting to make a new study and analysis examining the main parameters linked to prognosis in patients with CMH in this large cohort of 330 patients. PlosOne Editors will evaluate with interest this potential further study.**

Thank you for your suggestion. We have removed prognosis from the current study, and we will consider to publish it in a further study. **5) The most important and clinically relevant findings from this study are those linked to AL-Amyloidosis (i.e., the sensitivity and specificity of some parameters to detect a cardiac involvement in this systematic hematological disease). In fact, while patients with cardiac ATTR-Amyloidosis are easily diagnosed with bone scintigraphy (planar and/or SPECT), the cardiac involvement in AL-Amyloidosis is challenging (i.e., SPECT does not provide any diagnostic help). In addition, the role of CMR in AL-Amyloidosis remains uncertain. Hence, the authors should make focus in patients with AL-Amyloidosis.**

Thank you for your comment. We have made changes in the introduction and discussion section in order to direct the focus on the diagnosis of AL-amyloidosis.  **6) In the line with above-mentioned suggestion, the authors should significantly change the title, abstract, introduction, and discussion of the study, focusing only in the findings on the detection and diagnosis of cardiac AL-Amyloidosis. In this respect, a potential title would be “Potential Clinical Relevance of CMR to Diagnose Cardiac AL-Amyloidosis”. By the way, a potential abstract would be:**

**- Background: While patients with cardiac ATTR-Amyloidosis are easily diagnosed with bone scintigraphy (planar and/or SPECT), the detection of cardiac involvement in AL-Amyloidosis is challenging. In addition, the role of CMR in AL-Amyloidosis remains uncertain. Hence, the purpose of the present study was to analyze the potential role of CMR in the detection of cardiac involvement in patients with AL-Amyloidosis.
Methods: We included 35 patients with proved cardiac AL-Amyloidosis and two control groups constituted by 330 patients with HCM and 70 patients with arterial hypertension, who underwent CMR examination. Phenotype and amount of LV wall thickness, strain, and late gadolinium enhancement (LGE) were evaluated. Sensibility and specificity of several CMR parameters were analyzed comparing patients with cardiac AL-Amyloidosis vs those with CMH and vs. those with arterial hypertension.
Results: please describe the results of table 3 regarding AL-Amyloidosis vs CMH and vs. arterial hypertension.
Conclusions: The findings from this study suggest that septal myocardial nulling prior to blood pool nulling or difficulty in achieving myocardial nulling or septal diffuse subendocardial LGE proving excellent sensibility and specificity to determine cardiac involvement in patients with AL-Amyloidosis. Further larger studies are warranted to validate the findings from this study.**

Thank you for your suggestion. We have changed the title and the abstract of the article. We agree with the Editor that LGE CMR has an important role in the diagnosis of cardiac AL-amyloidosis. Moreover, we believe that our findings regarding the CMR-based strain analysis have also clinical significance and novelty. Therefore, besides LGE results, we would like to focus on strain, as well.

Full title: Potential clinical relevance of cardiac magnetic resonance including strain analysis to diagnose cardiac light chain amyloidosis.

Short title: CMR-based diagnosis of patients with cardiac amyloidosis.

Abstract:

Background: While patients with cardiac transthyretin amyloidosis are easily diagnosed with bone scintigraphy (planar and/or SPECT), the detection of cardiac involvement in light chain amyloidosis (CA) is challenging. Cardiac magnetic resonance (CMR) examinations have an essential role in the diagnosis of myocardial diseases; however, limited data are available from CMR-based feature-tracking strain analysis in this patient population. Hence, the purpose of the present study was to analyze the potential role of CMR in the detection of cardiac involvement in patients with light chain amyloidosis (CA).

Methods: We included 35 patients with proved CA and two control groups constituted by 330 patients with hypertrophic cardiomyopathy (HCM) and 70 patients with arterial hypertension (HT), who underwent CMR examination. The phenotype and degree of left ventricular (LV) hypertrophy, and the amount and pattern of late gadolinium enhancement (LGE) were evaluated. Global and regional LV strain parameters were calculated with feature-tracking strain analysis. Sensitivity and specificity of several CMR parameters were analyzed in diagnosing CA.

Results: The sensitivity of diffuse septal subendocardial LGE in diagnosing CA was 88% with a specificity of 99% when differentiating it from HCM and 100% when differentiating it from HT. The sensitivity and specificity of myocardial nulling prior to blood pool was 77% and 100%, respectively, both vs. HCM and vs. HT. Basal longitudinal strain had also high diagnostic accuracy (CA vs. HCM: sensitivity 69%, specificity 85%; CA vs. HT: sensitivity 94%, specificity 79%). CMR-based strain analysis was applicable for detecting apical sparing in CA patients.

Conclusions: The findings from this study suggest that CMR has high diagnostic relevance in the diagnosis of CA. Besides the excellent sensitivity and specificity of diffuse subendocardial LGE pattern and abnormal contrast kinetics, CMR-based strain analysis has also an important role in differentiating CA from HCM and from controls with HT. **7) Please do not add the symbol % next to the parameters’ description, but next to the values of sensibility and specificity.**

Thank you for your advice. We have added the symbol % next to the values instead of the parameters’ description. **8) Please add to the table 3 a file with the accuracy value.**

Thank you for your advice. We added the AUC values to the table 3.

**9) Please provide mandatorily the following cases/figures examples:
1- AL-Amyloidosis with septal myocardial nulling prior to blood pool nulling or difficulty in achieving myocardial nulling
2- Arterial hypertension without septal myocardial nulling prior to blood pool nulling or difficulty in achieving myocardial nulling
3- CMH without septal myocardial nulling prior to blood pool nulling or difficulty in achieving myocardial nulling
4- AL-Amyloidosis with septal diffuse subendocardial LGE
5- Arterial hypertension without septal diffuse subendocardial LGE
6- CMH without septal diffuse subendocardial LG**

Thank you for your suggestion. On Figure 3, we show a representative case of HCM without septal myocardial nulling prior to blood pool nulling or difficulty in achieving myocardial nulling and without septal diffuse subendocardial LGE (A), cases of CA with myocardial nulling prior to blood pool nulling (B) and diffuse subendocardial LGE (C), and a case of arterial hypertension without septal myocardial nulling prior to blood pool nulling or difficulty in achieving myocardial nulling and without septal diffuse subendocardial LGE (D). **10) Please provide a table comparing and describing the findings of the present study vs previous similar studies (i.e., role of CMR in AL-Amyloidosis; please see** [**https://pubmed.ncbi.nlm.nih.gov/?term=light%20chain%20(AL)%20amyloidosis%20AND%20diagnosis%20AND%20CMR&sort=date&page=4**](https://pubmed.ncbi.nlm.nih.gov/?term=light%20chain%20(AL)%20amyloidosis%20AND%20diagnosis%20AND%20CMR&sort=date&page=4)

Thank you for your advice. Recently a meta-analysis was published investigating the diagnostic performance of CMR in cardiac amyloidosis based on all relevant studies, thus we decided to refer to this article, as we cannot perform a more detailed analysis regarding this topic. **11) Please discuss previous similar studies in the discussion section.**

Thank you for your suggestion. We have completed the discussion section with the followings:

“A recent meta-analysis based on 18 published studies included 1,108 CA patients (69% were AL) and 907 control subjects, estimated a sensitivity and specificity of 84% and 80%, respectively, for LGE in diagnosing CA (21). According to another meta-analysis of 7 studies, the sensitivity and specificity of LGE CMR in diagnosing CA were 85% and 92%, respectively (22). Expert consensus recommendations (17,18) state that CMR has a central role in the non-invasive diagnosis of CA referring to several studies in which typical LGE pattern has been shown to have a diagnostic sensitivity of 85% to 90% (23–27).”
