# Supplementary material for: Potential clinical relevance of cardiac magnetic resonance to diagnose cardiac light chain amyloidosis
Source: PLoS One. 2022 Jun 13;17(6):e0269807. doi: 10.1371/journal.pone.0269807 (PMC9191721; doi:10.1371/journal.pone.0269807)
Supplement: Attachment — Submitted filename: Response to Reviewers.docx [file pone.0269807.s007.docx]

**Cover letter, questions and our responses (the questions are shown in bold):**

Dear Daniel A. Morris,

Thank you for your response to our manuscript. Attached, please find our revised manuscript. We have answered the questions that the Editor raised. We appreciate the helpful comments from the Editor. We believe that the manuscript has been improved by incorporating these comments and recommendations.

Thank you very much for your consideration. We look forward to hearing your decision.

Best regards,

Hajnalka Vago

Editor’s comment:

Thank you very much for your time in addressing all suggested revisions. In effect, the paper has significantly improved and it is still only some minor revisions to get the final version of this interesting study.

Pending Minor Revisions:

**1) Please use the term Al-Amyloidosis to refer to light chain amyloidosis. Hence, please use in the whole manuscript as well in the abstract and conclusion the term “cardiac AL-Amyloidosis”.**
Thank you for your suggestion. We have changed the term CA to cardiac AL-amyloidosis.

**2) Please make focus in the results section of the abstract and in the conclusion of the manuscript on the main findings of the study such as the high sensitivity and specificity of diffuse septal subendocardial LGE and myocardial nulling to differentiate cardiac AL-Amyloidosis from HCM and from HT as well as the low sensibility of a reduced GLS and of apical sparing to differentiate cardiac AL-Amyloidosis from HCM and from HT. Moreover, please highlight that a septum ≥ 14 mm has high sensitivity and specificity to differentiate cardiac AL-Amyloidosis from HT.**

Thank you for your advice. We have changed the results section of the abstract as follows:

“The sensitivity of diffuse septal subendocardial LGE in diagnosing cardiac AL-amyloidosis was 88% with a specificity of 99% vs. HCM and 100% vs. HT. The sensitivity and specificity of myocardial nulling prior to blood pool was 77% and 100%, respectively, both vs. HCM and vs. HT. Maximal wall thickness with a cut-off of 14 mm had high diagnostic accuracy when differentiating cardiac AL-amyloidosis from HT: sensitivity 94%, specificity 89%. The sensitivities of reduced global longitudinal strain plus apical sparing were low with high specificities. CMR-based strain analysis was applicable for detecting apical sparing in cardiac AL-amyloidosis patients.”

**3) The results on the diagnostic performance of LV basal segmental strain are additional or secondary findings given the high variability and low reproducibility of basal strain values in the LV. Hence, the manuscript should not be focused or centered on these findings and these findings should not be shown in the abstract or conclusion section.**

Thank you for your advice. We have deleted the results regarding basal strain values from the abstract. Our results of interobserver analyses show that intraclass correlation of basal LS was good, near to excellent (0.87), the intraclass correlation of basal CS was excellent (0.92) (S1 Table). Thus we believe that the results regarding basal strain values are relevant enough to be included in the manuscript beside the main findings.

**4) In line with the above-mentioned comments, please re-edit the title of the study as “Potential clinical relevance of cardiac magnetic resonance to diagnose cardiac light chain amyloidosis”.**

Thank you for your suggestion, we have changed the title as requested.
