# Supplementary material for: Potential clinical relevance of cardiac magnetic resonance to diagnose cardiac light chain amyloidosis
Source: PLoS One. 2022 Jun 13;17(6):e0269807. doi: 10.1371/journal.pone.0269807 (PMC9191721; doi:10.1371/journal.pone.0269807)
Supplement: Attachment — Submitted filename: Response to Reviewers.docx [file pone.0269807.s008.docx]

Additional Editor Comments:

1) Thank you very much for your efforts to improve the manuscript, which in fact has significantly improved. However, it remains the same issues as in the previous submissions. In this respect, the findings on the role of basal LS and CS to differentiate AL-Amyloidosis from CMH and HT are not clinically relevant. In this respect, a cutoff of basal LS at 21 and 16% and basal CS at 31% are within the range of normality, and thus, these cutoffs have low specificity (i.e., in other words, a high proportion of healthy subjects have values of basal LS about 16% and 21% and basal CS about 31% or even lower). In addition, the variability day to day of basal LS and basal CS is well known in clinical practice. Hence, as it has been stated and highlighted in previous submissions, the findings of the diagnostic performance of basal LS and CS or any strain parameter are of low clinical relevance to differentiating Al-Amyloidosis from CMH or HT and thus, these findings should play a secondary role in the manuscript.
2) Taking into consideration the above-mentioned issues please re-edit and refine the abstract, conclusions, and the first paragraph of the discussion section.
3) Please consider using an abstract like this:
- Background: While patients with cardiac transthyretin amyloidosis are easily diagnosed with bone scintigraphy, the detection of cardiac light chain (AL) amyloidosis is challenging. Cardiac magnetic resonance (CMR) analyses play an essential role in the differential diagnosis of cardiomyopathies; however, limited data are available from cardiac AL-Amyloidosis. Hence, the purpose of the present study was to analyze the potential role of CMR in the detection of cardiac AL-amyloidosis.
Methods: We included 35 patients with proved cardiac AL-amyloidosis and two control groups constituted by 330 patients with hypertrophic cardiomyopathy (HCM) and 70 patients with arterial hypertension (HT), who underwent CMR examination. The phenotype and degree of left ventricular (LV) hypertrophy and the amount and pattern of late gadolinium enhancement (LGE) were evaluated. In addition, global and regional LV strain parameters were also analyzed using feature-tracking techniques. Sensitivity and specificity of several CMR parameters were analyzed in diagnosing cardiac AL-amyloidosis.
Results: The sensitivity and specificity of diffuse septal subendocardial LGE in diagnosing cardiac AL-amyloidosis was 88% and 100%, respectively. Likewise, the sensitivity and specificity of septal myocardial nulling prior to blood pool was 71% and 100%, respectively. In addition, a maximal LV end-diastolic septal wall thickness ≥ 14 mm had an optimal diagnostic performance to differentiate cardiac AL-amyloidosis from HT (sensitivity 91%, specificity 83%). On the other hand, a reduced global LV longitudinal strain (< 15%) plus apical sparing had a very low sensitivity (6%) in detecting AL-Amyloidosis, but with very high specificity (100%).
Conclusions: The findings from this study suggest that CMR could have an optimal diagnostic performance in the diagnosis of cardiac AL-amyloidosis. Hence, further larger studies are warranted to validate the findings from this study.

4) Please consider using a conclusion like this:
- The findings from this study suggest that CMR could have an optimal diagnostic performance in the diagnosis of cardiac AL-amyloidosis. In this respect, the sensitivity and specificity of diffuse septal subendocardial LGE in diagnosing cardiac AL-amyloidosis was 88% and 100% and of septal myocardial nulling prior to blood pool was 71% and 100%, respectively. In addition, a maximal LV end-diastolic septal wall thickness ≥ 14 mm had an optimal diagnostic performance to differentiate cardiac AL-amyloidosis from HT (sensitivity 91%, specificity 83%). On the other hand, a reduced global LV longitudinal strain (< 15%) plus apical sparing had a very low sensitivity (6%) in detecting AL-Amyloidosis, but with very high specificity (100%). Hence, further larger studies are warranted to validate the potential key role of CMR in the diagnosis of cardiac AL-amyloidosis.

5) Please consider using in the first sentence and paragraph of the discussion section the following statements:
- Analyzing a cohort of 35 patients with proved cardiac AL-amyloidosis and two control groups constituted by 330 patients with hypertrophic cardiomyopathy (HCM) and 70 patients with arterial hypertension (HT), who underwent CMR examination, the findings from this study suggest that CMR could have an optimal diagnostic performance in the diagnosis of cardiac AL-amyloidosis. In this respect, the sensitivity and specificity of diffuse septal subendocardial LGE and of septal myocardial nulling prior to blood pool in diagnosing cardiac AL-amyloidosis was excellent. In addition, a maximal LV end-diastolic septal wall thickness ≥ 14 mm had an optimal diagnostic performance to differentiate cardiac AL-amyloidosis from HT. On the other hand, a reduced global LV longitudinal strain (< 15%) plus apical sparing had a very low sensitivity (6%) in detecting AL-Amyloidosis, but with very high specificity (100%).

Authors’ answer:

We thank the editor for his thorough work and detailed suggestions for improving the manuscript. We have changed the abstract, the conclusion, and the first sentence and paragraph of the discussion section as requested. We only deviated from the editor's proposal on one point: Data show that a LV end-diastolic septal wall thickness ≥ 15 mm had better diagnostic performance to differentiate cardiac AL-amyloidosis from HT than a cut-off of 14 mm (15 mm: sensitivity 91%, specificity 89%, AUC 0.900, 14 mm: sensitivity 91%, specificity 83%, AUC 0.870). However, if maximal end-diastolic wall thickness is analysed (not necessarily septal), the optimal cut-off is 14 mm (sensitivity 94%, specificity 89%, AUC 0.927). Since the maximal end-diastolic wall thickness ≥ 14 mm had the highest diagnostic performance of the above mentioned parameters, we decided to highlight it. Max. EDWT has also been added to tables 2 and 3.
