# Supplementary material for: Potential clinical relevance of cardiac magnetic resonance to diagnose cardiac light chain amyloidosis
Source: PLoS One. 2022 Jun 13;17(6):e0269807. doi: 10.1371/journal.pone.0269807 (PMC9191721; doi:10.1371/journal.pone.0269807)
Supplement: Attachment — Submitted filename: Response to Reviewers.docx [file pone.0269807.s009.docx]

Journal Requirements:

Please review your reference list to ensure that it is complete and correct. If you have cited papers that have been retracted, please include the rationale for doing so in the manuscript text, or remove these references and replace them with relevant current references. Any changes to the reference list should be mentioned in the rebuttal letter that accompanies your revised manuscript. If you need to cite a retracted article, indicate the article’s retracted status in the References list and also include a citation and full reference for the retraction notice.

Authors’ answer:

We have reviewed the reference list and made no changes.

Additional Editor Comments:
I would like to congratulate again to the authors for the effort made to improve this interesting study. In fact, the study is of high interest for the Journal and it will be for all medical community, given the originality the findings regarding the potential usefulness of CMR in the diagnosis of cardiac Al-Amyloidosis.
Just one last minor correction is pending. In this respect, please delete in the text, abstract, conclusions, and tables the “new” analysis titled “maximal LV end-diastolic wall thickness” since in clinical practice almost never are measured all walls thickness but only the septal and posterior wall thickness, which are easy correlated with the measurements from echocardiography. By the way, as the authors have been stated, please change / correct in the abstract, conclusion, and first paragraph of the discussion section the sentence “In addition, a maximal LV end-diastolic wall thickness ≥ 14 mm had an optimal diagnostic performance to differentiate cardiac AL-amyloidosis from HT (sensitivity 94%, specificity 89%)” by “In addition, a LV end-diastolic septal wall thickness ≥ 15 mm had an optimal diagnostic performance to differentiate cardiac AL-amyloidosis from HT (sensitivity 91%, specificity 89%)”.

Authors’ answer:

We thank again the editor for his thorough work and detailed suggestions for improving the manuscript. We have changed the abstract, the conclusion, and the first sentence and paragraph of the discussion section as requested. We have deleted max. EDWT in tables 2 and 3.
